# How to embed qualitative research in trials: insights from the feasibility study of the SAFER trial programme

**DOI:** 10.1186/s13063-022-06308-7

**Published:** 2022-05-12

**Authors:** Alison Powell, Sarah Hoare, Rakesh Modi, Kate Williams, Andrew Dymond, Cheryl Chapman, Simon Griffin, Jonathan Mant, Jenni Burt

**Affiliations:** 1 The Healthcare Improvement Studies Institute, University of Cambridge, Clifford Allbutt Building, Cambridge Biomedical Campus, Cambridge, CB2 0AH UK; 2 Primary Care Unit, University of Cambridge, Strangeways Research Laboratory, Worts Causeway, Cambridge, CB1 8RN UK; 3 Primary Care Unit, Department of Public Health and Primary Care, School of Clinical Medicine, University of Cambridge, Cambridge, CB2 0SP UK; 4 MRC Epidemiology Unit, Institute of Metabolic Science, School of Clinical Medicine, University of Cambridge, Cambridge, CB2 0SL UK

**Keywords:** Qualitative research with trials, Atrial fibrillation, Frameworks, Working relationships

## Abstract

Qualitative research can enhance the design, conduct and interpretation of trials. Despite this, few trials incorporate qualitative methods, and those that do may not realise their full potential. In this commentary, we highlight how qualitative research can contribute to the design, conduct and day-to-day running of a trial, outlining the working arrangements and relationships that facilitate these contributions. In doing so, we draw on (i) existing frameworks on the role of qualitative research alongside trials and (ii) our experience of integrated qualitative research conducted as part of the feasibility study of the SAFER trial (Screening for Atrial Fibrillation with ECG to Reduce stroke), a cluster randomised controlled trial of screening people aged 70 and above for atrial fibrillation in primary care in England. The activities and presence of the qualitative team contributed to important changes in the design, conduct and day-to-day running of the SAFER feasibility study, and the subsequent main trial, informing diverse decisions concerning trial documentation, trial delivery, timing and content of measures and the information given to participating patients and practices. These included asking practices to give screening results to all participants and not just to ‘screen positive’ participants, and greater recognition of the contribution of practice reception staff to trial delivery. These changes were facilitated by a ‘one research team’ approach that underpinned all formal and informal working processes from the outset and maximised the value of both qualitative and trial coordination expertise. The challenging problems facing health services require a combination of research methods and data types. Our experience and the literature show that the benefits of embedding qualitative research in trials are more likely to be realised if attention is given to both structural factors and relationships from the outset. These include sustained and sufficient funding for qualitative research, embedding qualitative research fully within the trial programme, providing shared infrastructure and resources and committing to relationships based on mutual recognition of and respect for the value of different methods and perspectives. We outline key learning for the planning of future trials.

**Trial registration:** Screening for atrial fibrillation with ECG to reduce stroke ISRCTN16939438 (feasibility study); Screening for atrial fibrillation with ECG to reduce stroke – a randomised controlled trial ISRCTN72104369.

## Background

Much has been written about the multiple benefits qualitative research can bring to the design, conduct and interpretation of trials. At the pre-trial stage, qualitative approaches can contribute to the optimisation of the content, quality, delivery and acceptability of the trial intervention [1–6]. Methods such as interviews and focus groups can explore patients’ experiences and beliefs [7–10] to inform the choice of important outcomes for trials and minimum standards for interventions. Such preparation may “reduce unwelcome surprises and costly errors during the main trial” ([4]: 257). Qualitative appraisal of the nature of obtaining and sustaining informed consent [2, 11, 12] can strengthen the ethical foundation of trials. Examination of issues that impair the efficiency and acceptability of recruitment processes can enhance trial uptake [2, 13–15] and participant retention. Crucially, qualitative methods (notably qualitative process evaluations which include ethnographic observations) can support the implementation of complex interventions by exposing the contexts and the social and behavioural processes that may derail or amplify their success [9, 16–21]. Qualitative research, then, may enhance and strengthen trials in manifold ways [2, 22, 23]. As Greenhalgh et al. comment [24]: “Few research topics in clinical decision making and patient care can be sufficiently understood through quantitative research alone.”

Despite these enticing sounding claims, in practice, very few trials incorporate qualitative methods. A review of three trial registers from 1999 to 2016 found only 0.03 to 3.4% of trials reported using qualitative methods; trials of complex interventions were the most likely to do so [7]. Only 2% of funded trial proposals in the UK from 2001 to 2010 described embedded qualitative research [25]. Even when qualitative research is included in trials, concerns have been raised about its quality, visibility and reporting [7, 20, 25, 26]. Such studies typically rely on standard methods like interviews and focus groups, rather than generating insights using the whole range of available qualitative methods [22], and are usually concerned with highly visible trial mechanisms (such as the content and delivery of interventions), rather than with wider trial processes [23]. As one paper summarised, qualitative approaches in trials “continue to be underused, underreported, inadequately described and contain systematic and methodological shortcomings” ([4]: 257).

Even when there is a commitment to the inclusion of qualitative approaches within a trial, there is little practical guidance on how to achieve this effectively. Debate about the philosophical appropriateness of integrating qualitative and quantitative data continues [27, 28], whilst concrete examples of integration are scarce [29]. A number of frameworks outline the potential contribution of qualitative research to trials [30]. Although these frameworks vary in their details, they typically use one of three lenses [31]: (i) a temporal focus on qualitative methods to use before, during and after a trial [32, 33]; (ii) a process-outcome evaluation framework with qualitative research used to explore components like the context, reach and implementation of an intervention [26, 31]; and (iii) a broader focus on how qualitative methods can contribute across varying aspects of a trial, including the intervention, trial design and context, outcomes, process and outcome measures, understanding of the target health condition and experience of the participants [8, 30, 34]. A further dimension included in some frameworks is the degree of integration between the qualitative research and the trial [6, 35].

This continued focus on abstract design issues hides a surprisingly under-developed literature on how to deliver high-quality qualitative research alongside trials, and good examples remain rare [36, 37]. A crucial absence is recognition of the role of interpersonal dynamics within the research team [38]. Too often, research aspirations are derailed by mundane practicalities or poor relationships [39]. A key question for all trial programmes therefore, alongside how to integrate qualitative and quantitative research, is how to integrate qualitative and quantitative researchers. Effective team working is too often taken for granted, only brought to the fore when studies encounter serious problems [23]. One existing framework highlights three types of joint working between trial and qualitative researchers: at best interdisciplinary (representing close working), at worst dysfunctional and, somewhere in the middle, multidisciplinary (working in parallel or in sequence) [38]. What this looks like on the ground is far from clear.

This commentary starts to address these gaps. Its aim is to describe the changes to the design, conduct and day-to-day running of a trial arising from embedded qualitative research and to explore the working arrangements and relationships between qualitative and trial team researchers that facilitated these changes. We use as a case study our experience of working closely together, as qualitative and trial teams, in the feasibility study of the SAFER trial [40], a cluster randomised controlled trial (RCT) of screening for atrial fibrillation (AF) in primary care.

## Embedded qualitative research: a case study

**Fig. 1:**
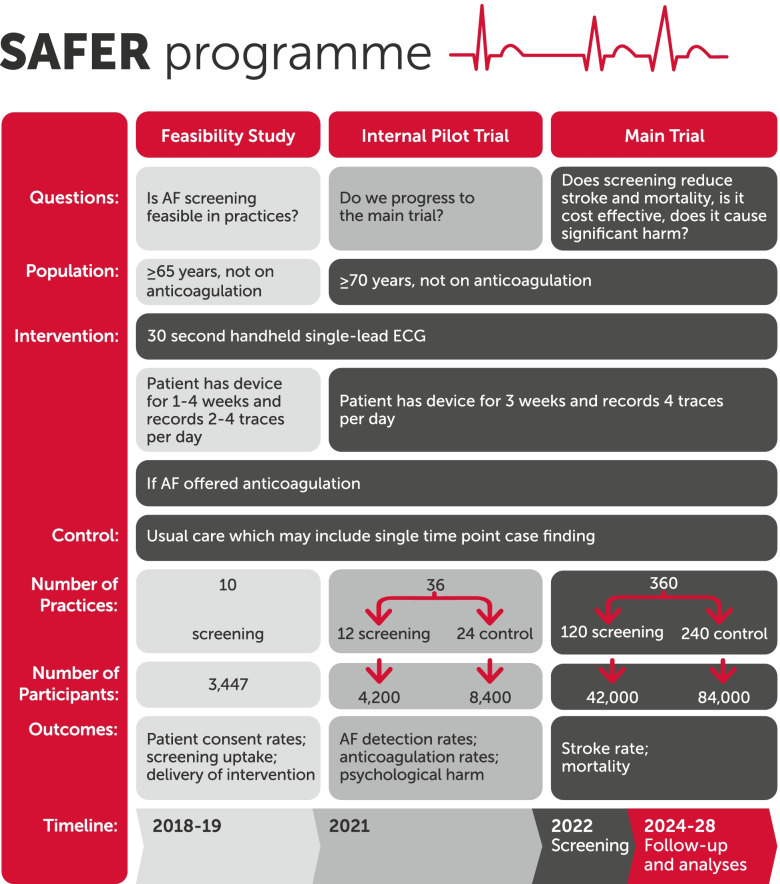
Outline of the SAFER programme. Note: a second small feasibility study (introduced in response to the COVID-19 pandemic) was carried out in 2020-2021 to assess the feasibility of remote delivery of the screening intervention

The SAFER trial is a large cluster randomised controlled trial of screening for the heart condition AF in older adults (age 70 and above) in primary care in England (Fig. 1). The main phase of the SAFER trial aims to recruit 126,000 consented patients from 360 general practices (84,000 patients in the control arm, 42,000 patients in the intervention or screening arm), with an average follow-up of 5 years; recruitment to the internal pilot began in spring 2021. The feasibility study on which this commentary is based was carried out in 2019 in ten general practices. These practices — all delivering the screening intervention in order to test feasibility — recruited a total of around 3440 participants from 8000 invitations.

The SAFER trial programme includes extensive, embedded qualitative research, running throughout all phases. In the feasibility study, this focused on exploring GP practice and participant experiences of AF screening. Data collection included longitudinal, repeat interviews with 24 screening participants (55 interviews in total); interviews with study invitees who declined to participate in the SAFER study (*n*=12); interviews with SAFER study participants who declined the offer of screening (*n*=12); interviews with staff and other stakeholders (*n*=19); and observations of screening appointments (*n*=47), along with observations of general care delivery, in three case study practices.

### Impact of qualitative research on the SAFER trial

In reflecting on the impact of qualitative research on the SAFER trial, we drew extensively on existing literature and used existing frameworks of qualitative research alongside trials, notably O’Cathain’s model of the stages at which such research may be utilised [34], to identify key areas of change.

We identified multiple modifications to the design, conduct and day-to-day running of the trial arising from the activities and presence of the qualitative team within the SAFER programme. The qualitative data and observations made by the SAFER qualitative team led to modifications within the intervention, the conduct of the trial and the timing and content of the assessment of outcomes (Table 1). Two significant changes were an alteration asking practices to notify all screening participants (not just ‘screen positive’ participants) of their screening results (discussed further below), and the inclusion of resources and information in recognition of the pivotal role played by practice reception staff in the trial delivery. As we outline below, a ‘one research team’ way of working is the norm on the SAFER programme. Trial team members emphasised that this way of working facilitated many and varied informal contributions to trial improvements as a result of the qualitative data and qualitative researchers. A key observation made at one of our joint discussions about our ways of working in preparation for writing this commentary was that there were “too many contributions to remember” and that it was hard to pull out specific examples because “this is so much part of the way we work”.

**Table 1:** Qualitative research contribution in the feasibility study of the SAFER trial programme

Aspect of the trial	Qualitative research contribution in the feasibility study of the SAFER trial programme	Example
Intervention		
Developing the intervention	-	-
Improving the intervention	✓	Informed decision to remove requirement to check heart rate parameters when a participant was set up with the screening device
Describing the intervention	✓	Described the form of the screening programme across different practices
Understanding how the intervention works: mechanisms of impact	✓	Identified drivers of screening uptake
Developing, refining or challenging theory	✓	Developed logic model of the intervention to inform programme theory
Understanding implementation	✓	Demonstrated the important and hidden role of practice receptionists in the trial
Exploring the feasibility of the intervention	✓	Highlighted practical aspects which impede patient and staff participation
Exploring the acceptability of the intervention	✓	Identified participant and health professional concerns about participants only receiving results if they had a ‘positive’ AF result
Understanding fidelity, reach and dose of the intervention	✓	Described the form of the screening programme across different practices
Identifying the value of the intervention	✓	Exposed patient and staff views on the importance of screening for and detecting AF
Identifying perceived benefits and harms	✓	Described benefits and harms, both those articulated by participants and those seen in observations
Understanding the context in which the intervention is tested	✓	Analysed practice differences which affect trial processes
Conduct		
Identifying effective and efficient recruitment practices	✓	Contributed to improvements in recruitment processes (e.g. revisions to participant information, enhanced media coverage)
Improving retention of participants	✓	Suggested improvements to participant information based on participant feedback Highlighted the importance of brief feedback and a thank you message to be shared with practices and patients
Maximising diversity	✓	Identified challenges for some participants in engaging and understanding trial material; suggested changes to participant documents to increase the clarity of the message
Understanding impact on participants, practitioners, and researchers of the RCT	✓	Suggested changes to staff training and documentation to reduce burden on staff
Undertaking an RCT that is acceptable	✓	Improved participant information around selection of participants
Improving ethical conduct	✓	Highlighted potential confusion from participant information about the length of the screening programme Reinforced participant information about action to take in the event of symptoms
Adapting RCT procedures to fit local contexts	-	-
Outcomes of the RCT		
Selecting outcomes important to patients and practitioners	-	-
Understanding variation in outcomes	✓	Identified contributory reasons for differential uptake of screening between patient groups
Measures in the RCT		
Identifying the accuracy of proposed measures	-	-
Improving completion of outcome measures	✓	Clarified requirements for staff completing CRFs (case report forms)
Developing outcome measures	✓	Informed the timing and content of harms of screening questionnaires
Understanding the health condition in the RCT	✓	Contributed to an understanding of the burden of AF for patients

The aspects of the trial listed in the first column of this table come from the ‘aspects of an RCT’ framework by O’Cathain and colleagues [30, 34]

### Routes to impact: the importance of integrated working arrangements and respectful relationships

One question we have reflected on extensively is what it was about the SAFER programme that made these contributions possible: what factors had enabled and enhanced the changes we identified? We considered the argument made by several authors (e.g. 25,26) that the degree of integration between qualitative research and a trial — the degree of ‘embeddedness’ of the qualitative research — was an important determinant of the extent to which qualitative research could broaden and sustain its contribution. This led us to reflect on something that we had largely taken for granted: the degree to which the qualitative research and qualitative researchers were an integral part of the SAFER programme. We examined the set-up, organisation and delivery of the SAFER programme and identified key aspects that underpinned this integration and that we believe enabled and enhanced the changes we identified.

#### Qualitative research workstreams were central and well-resourced

The trialists involved in the study were sensitised to the value of qualitative research through previous positive experience, both in general and particularly in relation to the topic of screening for AF. They were therefore keen to ensure that qualitative research was central to the SAFER endeavour from the outset. The senior qualitative lead contributed to the design of the programme from an early stage and was a co-investigator on all grant applications; additional senior qualitative collaborators were named on the main programme grant application. The senior qualitative lead was funded at 20% full-time equivalent (FTE) throughout the feasibility study and main trial, and a total of 7.5 years FTE was allocated to postdoctoral qualitative researchers. Senior members of the trial team sat on the appointment panels for these researchers.

The protocol made clear that the qualitative components of the trial were essential to fulfilling the trial objectives and meeting policy requirements. The SAFER research team set out to answer the overall research questions of the trial (in brief, the feasibility, harms, effectiveness and cost-effectiveness of a national screening programme for AF in primary care) by drawing on a range of types of data: clinical data, other quantitative data (e.g. engineering and organisational data), qualitative data and health economic data. Furthermore, the protocol explicitly referred to the need to *integrate* the qualitative findings with the quantitative trial outcomes in order to address the core aim of the trial and the potential implementation of a national screening programme after the trial.

#### A “one research team” approach was innate

Working arrangements promoted a ‘one research team’ approach from the outset, facilitated by the co-location of the SAFER qualitative research team in the same University department as the SAFER trial team. The structure of meetings within the programme reflected the integrated ‘one team’ approach: separate qualitative research team and trial team meetings that discussed detailed work processes, data collection and data analysis, fed into joint meetings attended by both. A key joint meeting was the monthly Trial Management Group meeting: trial team members and qualitative team members who were not part of the management group itself acknowledged the importance of attending this meeting in understanding the whole trial and how the different workstreams were inter-connected. Some of the meeting arrangements were set up at the outset and others emerged as the feasibility study progressed. Notably, regular smaller meetings between members of the trial team and the two postdoctoral qualitative researchers were established to ensure that the qualitative and quantitative data collection streams were co-ordinated effectively and informed by each other. This meeting was supported by a shared ‘living’ document, used as a focus for recording, discussing and addressing emerging issues.

The qualitative data collection processes were integrated into the trial ‘study coordination’ database. This was facilitated by holding meetings between the database manager, members of the qualitative research team and the trial team to design and modify the database. This integration within the database enabled qualitative and trial team members to work together to coordinate the involvement of selected trial participants in the qualitative strands of the study (e.g. in successive qualitative interviews at different points on the screening pathway) and to maintain shared records of communication with participants. A designated member of the trial team was responsible for supporting the administrative aspects of the qualitative fieldwork (e.g. contacting participants to arrange interview times).

Between meetings, research team members were in regular contact by phone, email and (later) a dedicated Microsoft Teams chat [41], covering work such as consulting on draft documents or debating potential changes to the trial protocol. All trial documents were available on shared servers so that they could be worked on or amended by a range of team members as appropriate. In addition to daily informal communication and reports made at joint meetings, the qualitative team wrote a rapid, formal report at the end of the feasibility study. It summarised emergent findings from the qualitative research, reported early analysis on patient and staff experiences in the trial and on how practices implemented it, and made observations for the team to consider in relation to the internal pilot and the main trial phases. The report was shared with the Programme Steering Committee members. The trial team took all these observations forward.

#### Diverse and complementary skills and experience were respected and used

Close working between members of the SAFER team meant that decisions were informed by diverse research insights and professional experience. Qualitative researchers on the team brought experience of designing qualitative studies and collecting, analysing and writing up qualitative data from patients, health professionals, managers and wider stakeholders. As individuals and as a team, they had an orientation to the research process that was different from, but complementary to, the orientations of the members of the trial team who came from other research and professional backgrounds. This could be seen in meetings when individuals drew on experience or data gained outside of or prior to the SAFER trial in explaining a point of view or raising a concern. Equally, the trial team were able to inform qualitative research plans when adjustments were needed to work around the needs of the trial processes.

#### A shared understanding of the purpose of the feasibility study was developed

Key to effective working as a team in all this was a shared understanding (and a willingness to act on this understanding) that the express purpose of the feasibility study was to learn from experience and to refine and improve the processes and documents in preparation for the main trial. This shared understanding helped to foster an openness to constructive suggestions from any team member and a readiness to do the additional work of amending trial documents and submitting often lengthy and detailed protocol amendments to serve that aim.

We describe here three examples of changes (included in Table 1) that resulted from this way of working.

##### Reporting screening results to all participants

Many changes to trial procedures emerged through repeated discussions amongst the SAFER team. One example emerged from an observation by the qualitative team of how screening results were being reported to participants by practices. Although the protocol stated that practices would only notify ‘screen positive’ participants of their screening results, one practice was contacting all participants with their screening results (positive or negative). Qualitative data showed that participants and practice staff had concerns that only returning ‘positive’ screening results risked provoking participant anxiety (e.g. that a positive screening result may have been ‘lost in the system’) and could be discourteous to trial participants. The issue was discussed further by the Trial Management Group, at an ‘All investigators’ meeting and in detail by the chief investigator and the qualitative lead to explore how the concerns might best be addressed within the constraints of funding. The trial team subsequently submitted a protocol amendment that screening practices would be encouraged to inform all participants of the result of their screening (positive or negative).

##### Providing a trial update to participants 

Another example of qualitative data leading to a change in trial processes concerned feedback to participants and practices. A recurrent finding was that trial participants were keen to know more about the trial and to receive feedback after their participation in addition to the option to receive a summary of the results at the end of the trial. The qualitative team suggested that this earlier feedback might also have the wider benefit of encouraging participants to engage with future research studies. The trial team agreed to produce a brief ‘feedback and thank you’ message from the feasibility study that practices could share with patients.

##### Removing the heart rate check from the screening device set up

The trial team also made specific requests for qualitative data to inform decisions on future trial processes. For example, a query was raised about whether it was desirable to include a heart rate check when a participant was set up with the screening device (i.e. to assess whether the participant’s heart rate was within a pre-specified range). This requirement was a barrier to designing a ‘remote’ screening programme to enable the trial to continue during the COVID-19 (coronavirus 2019) pandemic. The Trial Management Group reviewed both quantitative and qualitative data from the feasibility study to inform the decision. The qualitative team collated data from patient and practice staff interviews and from observations of screening appointments, which demonstrated that the heart rate check was a source of anxiety for some patients and provided false reassurance to others. It also created challenges for the staff running the screening clinic as it could generate further action (e.g. referral to the duty doctor). The trial team used the qualitative data together with the quantitative data (which showed that no important health conditions were picked up as a result of the heart rate check) to support removing the heart rate check.

As Table 1 illustrates, qualitative insights and researchers drove a number of changes to the SAFER trial following the feasibility study. Issues brought to the fore and ‘championed’ by the qualitative team ranged from high profile trial design considerations to mundane practicalities; cumulatively, these had the potential to improve the efficiency of conduct, experience of participating patients and practice staff, and the overall reputation of the trial and the institutions involved. The qualitative team acted as advocates both for the specific findings identified through qualitative data analysis and for the broader perspectives that a qualitative research orientation represents. It was intended from the outset that qualitative research would contribute to changes to the trial: the feasibility study was set up to explore aspects of trial design (e.g. acceptability to participants, viability in busy practices). The SAFER trial team also had in place mechanisms to receive feedback from practice and patient participants (e.g. device feedback forms from patients, case report forms from practice participants and teleconferences with practice participants to share experiences and tips for use by future practices in the trial). However, the trial benefitted not just from the qualitative data, but also from the additional and complementary resource of the qualitative *researchers*, who brought insights from their previous research and from the qualitative research within the SAFER programme to bear on a daily basis.

#### Senior investigators with expertise in qualitative and quantitative methods

The senior investigators were conversant with and supportive of both qualitative and quantitative methods. Thus, they were able to understand and appreciate the contribution of different forms of data and their interaction and to ‘translate’ or interpret those meanings to others. We are not suggesting that trial researchers need to be confident in using both qualitative and quantitative methods themselves. In our experience, this dual qualification is rare among health researchers and is unlikely to be necessary. What is needed is a team culture within which all members (regardless of discipline) respect and value the contribution of other methods and the knowledge and experience of other researchers on the team and that this culture is endorsed and embodied by the senior investigators on the trial.

In referring to a qualitative/quantitative dualism, we may risk evoking and even contributing to the long-running and ongoing debates about the existence of such a dualism and its ramifications. Nevertheless, there remains considerable evidence (e.g. 2,24,37) that whilst these debates may be old, the standpoints and methodological challenges that give rise to them do persist in some of the structures and influential discourses in health research.

### Challenges

The many benefits achieved did not occur without challenges. Proponents of qualitative research alongside trials acknowledge a range of potential difficulties. Notably, an embedded RCT places particular demands on the design of the trial, requiring careful consideration of the timing of data collection, analysis and reporting. Parallel workstreams must not impede each other or disadvantage participants. The timing and mechanisms of reporting (both internal and external) and of formal and informal communication between the researchers must be efficient and accommodating of trial processes [2, 37, 42]. An assessment needs to be made at the design stage as to whether any data collection streams risk ‘contaminating’ the outcomes from other parts of the trial [42, 43], and how to mitigate such risks. Recruitment of healthcare organisations and of individuals to the trial requires disclosure at the outset of the details of all elements of the trial (both quantitative and qualitative) and an indication of which elements are compulsory and which are optional. The scope for refining aspects of the qualitative data collection in response to emerging findings may therefore be more limited than in a discrete qualitative study.

One of the concerns expressed about the inclusion of qualitative approaches in an embedded RCT is that embedded designs may undervalue and underutilise interpretive qualitative approaches. Interpretive qualitative approaches are broadly concerned with interpreting the ways that actors in the study setting (e.g. healthcare professionals and patients participating in the trial) understand its activities, structures and language and the meanings they give to their experiences in the setting [8, 37]. Such approaches typically use qualitative research methods that are more time- and labour-intensive for researchers or participants (e.g. ethnographic observations, diary methods [26]). As we noted in the ‘Background’ section, qualitative research in trials has tended to use standard methods, such as interviews and focus groups, rather than the full range of qualitative approaches. This may well be because of the scheduling constraints imposed within an RCT design or because of limited funding allocated to the qualitative research elements. Our contention is that when the qualitative research is built into the design of the trial from the outset, with sufficient resourcing and qualitative expertise throughout, it is possible to enlarge the range of qualitative methods being used [43] and to give due weight to interpretive approaches.

The risk of selection bias poses a further challenge. Although this is a risk faced by all research studies to some degree, qualitative research carried out in an embedded RCT grapples with a particular challenge: by definition, the qualitative research participants are individuals who are willing and able both to engage with the overall trial and to additionally participate in qualitative research (e.g. taking part in interviews or being observed during healthcare appointments). Selection bias may also occur in the recruitment of healthcare organisations in embedded RCTs, such as towards those which are able to accommodate qualitative research data collection, e.g. by providing researchers with access to rooms or staff. These particular risks can be offset by designing the trial to include diverse participants and healthcare organisations, offering a range of data collection methods and recruiting to the qualitative research ‘non-standard’ participants (e.g. participants who decline the trial invitation or the trial intervention).

Threats to recruitment and retention in embedded RCTs can be reduced by patient and public involvement (PPI) in the design of the trial and the participant documentation, offering a range of data collection methods appropriate to the study population and ensuring that qualitative research participants are not disadvantaged or overburdened relative to other trial participants.

A further challenge concerns assessing whether any of the trial outcome measures may be compromised by embedded qualitative research [42], for example if research participants change their minds about trial participation or about the intervention as a result of taking part in interviews or focus groups or withdraw from the trial because of research fatigue.

Other considerations faced by embedded RCTs include the resource implications of labour-intensive qualitative research methods, issues of consent and confidentiality for trial participants, the possibility of unplanned modifications to the trial intervention during the full RCT and the risk that qualitative researchers may identify issues that disrupt existing processes and are challenging to address [8, 23, 31, 42, 43]. As Russell et al. noted in response to qualitative insights brought to a trial: “One conclusion that could be drawn from these results is that they unhelpfully complicate matters for those undertaking a trial” ([37]:4). To these challenges, we would add, from the perspective of the qualitative researchers: the constraints of working within the more bounded and often externally driven timelines of a trial (in contrast to the more fluid timescales and greater flexibility that may be possible within a discrete qualitative research study), the challenge of maintaining focus within a large and complex trial in which there are many processes and themes of potential interest, the need to communicate to trial team colleagues the breadth of the qualitative team interest, the ‘hidden’ work of contributing to routine trial processes and the subsequent impact on the social science research that is a major focus for the qualitative researchers, the need to cultivate an identity that is part of the team but able to act as ‘critical friends’ when appropriate and the risk that taking the lead on patient and public involvement (PPI) may locate it in ‘the qualitative domain’. From the perspective of the trial delivery team, the challenges included managing the complex processes of a large trial whilst simultaneously keeping a watching brief for possible issues of interest to bring to the attention of the qualitative researchers. A further challenge was to handle the practicalities of sequencing invitations to participants who were involved in both qualitative and quantitative aspects of the trial and ensuring that these arrangements did not disadvantage or overburden individuals or affect the trial processes. We do not want to over-emphasise these challenges. There is a tendency in the literature to place considerable weight on the challenges of including qualitative research in trials and very little on the converse: the substantial risks to trials that go ahead in the absence of a qualitative perspective. We turn now to offer some observations from our experience and from the literature for the conduct of future trials.

## Practical lessons for future trials

We believe that current methodological frameworks do not go far enough in recognising and describing the value of qualitative research and qualitative researchers to trials [8, 26, 30–33]. Such frameworks may even have the inadvertent effect of limiting the contribution of qualitative research to trials by ‘boxing off’ the scope of qualitative activities or by framing them in ways that are overly trial-centric. There is a risk that the qualitative research is simply bolted on to a trial’s original conception and aim, with limited power to infuse the trial as a whole. The many benefits of combining qualitative research with trials have led some authors (e.g. 22,31) to argue that a combination of quantitative and qualitative data should be the standard for all interventional studies. Guidance from research bodies like the Medical Research Council (MRC) in the UK [44] and the National Institutes of Health (NIH) Office of Behavioral and Social Sciences [45] in the USA suggests that using a combination of research methods is likely to enhance understanding of complex health situations and to reduce the disadvantages of relying on one type of data alone. We would agree that the challenging issues facing health services require multiple forms of data and therefore a combination of research methods to address them. However, studies need to be set up and carried out in a way that enables integrated ways of working. It is possible to design a trial that on paper looks as if it will enable the integration of qualitative and quantitative data but that in practice fails to realise the full benefit of the integration of qualitative and quantitative *researchers*: integration that allows for other benefits to emerge from working together. To realise the full potential of qualitative research in trials, future trial programmes should carefully consider how trial funding, structure and day-to-day working arrangements will enable and promote effective collaborative working. In Table 2, we distil practical points which our experience and the literature suggest can contribute to achieving this.

**Table 2:** How to embed qualitative research in a trial

• Ensure senior qualitative input as a co-applicant on the trial from the earliest stages [6] • Include qualitative researchers as full team members from the outset [6, 46] • Embed the qualitative research within the trial design, protocol and funding [25, 26] • Provide sufficient resources for the qualitative research: this is important for parity of esteem, high-quality robust qualitative research and meaningful input to the trial [2, 6] • Work from the assumption that both qualitative and quantitative data are essential for the trial and will feed into its development and conduct and its dissemination and eventual policy impact [6, 22, 26] • Where possible, locate the qualitative research within the same institution as the trial team (ideally with geographical proximity) • Establish regular meetings that enable the qualitative and quantitative researchers on the trial team to share the emerging findings and their implications for the trial, to coordinate data collection across the different workstreams and to develop a sense of a shared mission and shared goals [2, 6] • Encourage and facilitate informal daily interactions that support collaboration, sharing of information and robust processes [2] • Budget for infrastructure for shared IT provision (e.g. shared database management/development, shared secure servers for shared documents and databases) • Recognise and acknowledge separate roles, responsibilities and expertise of qualitative and trial team members but allow for some sharing of tasks where appropriate and helpful (e.g. to ease bottlenecks and meet deadlines) • Consider joint authorship of qualitative researchers and the trial delivery team on publications and other outputs	

This table combines key lessons from the literature (referenced) and insights from our practical experience on the SAFER trial

## Conclusions

Qualitative research can contribute in multiple ways to trials but this work cannot be done ‘off the side of the desk’. If the full benefit of qualitative research within trials is to be realised, qualitative research must be valued. This means that it needs to have equal status with the rest of the trial, to be well resourced and to be embedded in the trial’s structures and relationships.

## Data Availability

Not applicable.

## Abbreviations

AF: Atrial fibrillation
CRF: Case report form
COVID-19: Coronavirus 2019
ECG: Electrocardiogram
FTE: Full-time equivalent
MRC: Medical Research Council
NIH: National Institutes of Health
PPI: Patient and public involvement
RCT: Randomised controlled trial
SAFER: Screening for Atrial Fibrillation with ECG to Reduce stroke
